# Evaluation of bleaching agent effects on color and microhardness change of silver diamine fluoride-treated demineralized primary tooth enamel: An in vitro study

**DOI:** 10.1186/s12903-022-02371-3

**Published:** 2022-08-12

**Authors:** Azade Rafiee, Mahtab Memarpour, Hadi Benam

**Affiliations:** 1 Oral and Dental Disease Research Center, Department of Pediatric Dentistry, School of Dentistry, Shiraz University of Medical Sciences, Shiraz, Iran; 2 Student Research Committee, Dental School, Shiraz University of Medical Sciences, Shiraz, Iran

**Keywords:** Remineralization, Silver diamine fluoride, Potassium iodide, Microhardness, Color change, Scanning electron microscopy

## Abstract

**Background:**

The present study aimed to assess the impact of application of fluoridated- 10% carbamide peroxide (CP) with or without potassium iodide (KI) on silver diamine fluoride (SDF)-treated enamel surface in the primary teeth.

**Methods:**

After stained-remineralized caries lesions (s-RCLs) creation, 96 teeth were randomly allocated to four experimental groups: Group 1:SDF-treated enamel followed by 8-h/day application of 10% CP for 2 weeks; Group 2: SDF-treated enamel followed by 15-min/day application of 10% CP for 3 weeks; Group 3: SDF + KI-treated enamel followed by 8-h/day application of 10% CP for 2 weeks; and Group 4: SDF + KI-treated enamel followed by 15-min/day application of 10% CP for 3 weeks. Enamel microhardness (EMH) test (n = 12) and spectrophotometric color assessment (n = 12) was performed at four stages: baseline (intact enamel), demineralized enamel, aged remineralized-stained enamel, and after final intervention. Sixteen samples were used for SEM evaluation. Data were analyzed with the paired t-test, one-way ANOVA, and Tukey’s post-hoc test (*p* < 0.05).

**Results:**

EMH values in all groups showed significant decrease after demineralization (all, *p* < 0.00001). All samples showed complete recovery of EMH values (%REMH) after SDF application compared to demineralization (%REMH_SDF_) (*p* = 0.971). Bleaching caused a slight decrease in %REMH for all groups. However, the differences were not statistically significant (*p* = 0.979). SEM findings revealed no changes in enamel porosity after bleaching. Bleaching application ameliorated the discoloration in all groups (all, *p*** < **0.00001). All samples in Groups 2 and 4 had significantly lighter color after 21 days as compared to 14-day exposure to the bleaching material (both, *p* < 0.00001).

**Conclusions:**

SDF application on demineralized primary tooth enamel completely recovered enamel microhardness. 10% carbamide peroxide effectively bleached SDF stain without causing significant decrease in EMH values. Color improvement was more evident with the use of KI immediately after SDF application. Both 15-min and 8-h application of fluoridated CP resulted in statistically similar color enhancement in primary teeth.

**Supplementary Information:**

The online version contains supplementary material available at 10.1186/s12903-022-02371-3.

## Introduction

Dental caries is a biofilm-mediated disease, characterized by dynamic episodes of demineralization and remineralization that deteriorates the tooth structure. The current caries control strategy consists of hindering the progression of lesions and promoting remineralization [1]. Fluoride compounds are the most commonly used materials for preventing and arresting dental caries [2]. The use of professionally applied fluoride materials such as acidulated phosphate fluoride (APF), sodium fluoride gel and varnish, and silver diamine fluoride (SDF) is a cost-effective and non-invasive modality [3].

Silver diamine fluoride (SDF) can remineralize tooth structure and arrest dental caries by formation of silver phosphate and calcium fluoride precipitates, and disruption of cariogenic microorganisms [4]. Various concentrations of SDF solution are commercially available, ranging from 10 to 38% [5]. However, its application causes permanent black staining on the porous tooth structure. The use of potassium iodide solution (KI) immediately after SDF application decreases the availability of silver ions by forming yellowish deposits of silver iodide, thus, reducing the degree of tooth staining [6].

Treatment options regarding esthetic enhancement of stained-remineralized caries lesions (s-RCLs) are few and mostly limited to dental microabrasions and restorations [7]. It is proposed that dental bleaching might help ameliorate these discolorations [7]. Many studies with controversial results have assessed the effect of bleaching products on mineral content and physical properties of the tooth surface [8–12]. Since the acidic nature of the whitening products raised concerns regarding the possibility of alteration in the enamel composition, fluoridated dental bleaching products were introduced to the market [13]. Studies have reported the remineralization potential of these fluoridated dental bleaching products [13, 14]. There are few studies, mostly case reports, to evaluate the use of bleaching agents in primary teeth [15–20]. Ten percent carbamide peroxide is the safest and the most effective bleaching material to be used in children, even in the existence of mild caries [15, 21].

Studies to evaluate the efficacy of bleaching agents on s-RCLs in the permanent dentition are limited [7, 22–25]. To the authors’ knowledge, this is the first study on this topic in the primary teeth. Therefore, this study aimed to design an in vitro model to create s-RCLs and to test the impact of duration of application of fluoridated- 10 percent carbamide peroxide with or without KI on surface microhardness, enamel morphology, and color change.

## Methods

### Study design

Totally, 118 caries-free primary anterior teeth, extracted due to orthodontic treatment, were collected based on a protocol approved by the Ethics Review Committee of Shiraz University of Medical Sciences. Written informed consents for the use of the teeth were obtained. After removing the roots to the level of one mm under the cementoenamel junction, the specimens went through washing, disinfecting by immersion in 0.1% chloramine T solution for one month, and storing in a weekly renewed deionized water at 37 °C until use. Prior to the beginning of the experiment, the samples were assessed under a stereomicroscope (×10) to exclude teeth with cracks, anomalies, stains, or defects. 104 teeth fulfilled the selection criteria.

### Sample preparation

To prepare enamel blocks, the crowns were embedded in acrylic resin with the labial surface parallel to the mold. Each tooth surface was serially polished with 600-, 800-, 1200-, 2400-, and 4000-grit waterproof silicon carbide paper followed by 1-μm aluminum oxide to obtain a horizontal and smooth surface. Next, the samples were washed for 20 s in distilled water, dried, and covered with two layers of nail polish except for a 2 × 4 mm window on the flattest portion of the enamel surface.

### Stained-remineralized caries-like lesions creation

To create early caries lesions, each block was demineralized at 37 °C for 96 h in 15 mL of the demineralizing solution containing 0.1 mM lactic acid solution, 3 mM CaCl_2_, 3 mM KH_2_PO_4_, and 0.2% guar gum. The final pH was adjusted to 4.5 using 50% sodium hydroxide [26]. The solution was refreshed after 48 h. At the end of the fourth day, each sample was washed with deionized water for 20 s and allowed to air dry.

For s-RCLs creation, we applied 38% SDF solution (Caries arrest, Dengen dental, India) to all exposed enamel surfaces and agitated the solution with a micro-brush for 1 min. After 2 min, the excess and unreacted SDF was blotted with a cotton pellet.

### Aging of the specimens

To better simulate the oral cavity environment, the samples underwent a thermocycling aging procedure for 1000 cycles at the temperatures between 5 and 55 °C with a dwell time of 30 s and a transfer time of 15 s. The SDF-treated samples were left in deionized water for 2 weeks.

### Group allocation

Of 104 selected teeth, 8 samples were used for scanning electron microscopy (SEM) evaluation at baseline (n = 2), after demineralization (n = 2), after SDF application (n = 2) and after SDF + KI application (n = 2). The remaining 96 teeth were randomly divided into four experimental groups (n = 24) based on the bleaching protocol and time using random allocation software version 2.0. The samples in each group were arbitrarily divided into two sub-groups to assess enamel microhardness (EMH) (n = 12) and color change (n = 12).

#### Group 1: 10% CP 8 h/day for 14 days

Ten percent fluoridated-carbamide peroxide (CP) (pH 6.5; Opalescence®, Ultradent Products, Inc., USA) was applied on the enamel surface of each specimen (1 mm thick) and kept for 8 h at 37 °C. Then, the samples were rinsed with running deionized water for 20 s to remove the bleaching agent and left in the deionized water for the remainder of the day to prevent desiccation. This cycle was repeated for 14 days.

#### Group 2: 10% CP 15 min/day for 21 days

Ten percent CP (pH 6.5; Opalescence®, Ultradent Products, Inc., USA) was applied on the enamel surface of each specimen (1 mm thick) for 15 min at 37 °C as explained for Group 1. This cycle was repeated for 21 days.

#### Group 3: 10% CP 8 h/day for 14 days on SDF + KI-treated enamel

A generous amount of 10% potassium iodide (KI) solution was immediately applied after SDF application using a microbrush. This procedure was repeated until the creamy white color precipitate turned clear. Then the intervention was performed as explained for Group 1.

#### Group 4: 10% CP 15 min/day for 21 days on SDF + KI-treated enamel

After KI application, the intervention was performed as explained for Group 2.

### Microhardness test

Twelve samples in each group underwent the Vickers microhardness test at four stages: baseline (intact enamel), demineralized enamel, aged remineralized-stained enamel (SDF-/ SDF + KI-treated surfaces), and after bleaching. For Groups 2 and 4, the microhardness values were also recorded at day 14. For EMH evaluation, a Vickers diamond indenter (MHV-1000Z, SCTMC, China) was used at 200 g force for 15 s, at five points of 100 µm distance per sample. The percentage recovery of enamel microhardness (%REMH) after s-RCLs creation and the final exposure to bleaching material was determined as follows:

$$\begin{aligned} \% {\text{REMH}}_{{{\text{SDF}}}} & = \left( {{\text{VHN}}_{{{\text{SDF}}}} {-}{\text{VHN}}_{{{\text{Demineralization}}}} } \right) \\ & \quad /\left( {{\text{VHN}}_{{{\text{Baseline}}}} {-}{\text{VHN}}_{{{\text{Demineralization}}}} } \right) \times {1}00 \\ \end{aligned}$$

$$\begin{aligned} \% {\text{REMH}}_{{{\text{2 - week int}}.}} & = \left( {{\text{VHN}}_{{\text{2 - week intervention}}} {-}{\text{VHN}}_{{{\text{Demineralization}}}} } \right) \\ & \quad /\left( {{\text{VHN}}_{{{\text{Baseline}}}} {-}{\text{VHN}}_{{{\text{Demineralization}}}} } \right) \times {1}00 \\ \end{aligned}$$

$$\begin{aligned} \% {\text{REMH}}_{{{\text{3 - week int}}.}} & = \left( {{\text{VHN}}_{{\text{3 - week intervention}}} {-}{\text{VHN}}_{{{\text{Demineralization}}}} } \right) \\ & \quad /\left( {{\text{VHN}}_{{{\text{Baseline}}}} {-}{\text{VHN}}_{{{\text{Demineralization}}}} } \right) \times {1}00 \\ \end{aligned}$$

### Surface morphology assessment

At the end of the intervention, two samples out of the 12 samples of the colorimetric assessment in each group, were randomly selected for SEM evaluation (n = 8). As explained before, we also prepared extra 8 enamel blocks with 2 × 4 mm window for microscopic assessment at baseline (n = 2), after demineralization (n = 2), after SDF application (n = 2) and after SDF + KI application (n = 2). The selected 16 samples were dehydrated with series of ethanol solutions and sputter-coated with gold in a vacuum evaporator. The surface morphology was examined with SEM (VEGA, Tescan, Brno, Czech Republic) at 20 kV in high-vacuum mode and 1500× magnification.

### Color assessment

L*a*b* values (Commision Internationale de l’Eclairage) of each sample was acquired for each specimen at baseline (intact enamel), demineralized enamel, aged remineralized-stained enamel (SDF-/ SDF + KI-treated surfaces), and after final exposure to bleaching material. For groups 2 and 4, the L*a*b* values were also recorded at day 14. L* value expresses brightness as numbers from 0 (dark) to 100 (bright), a* value describes redness (+ a*) to greenness (− a*), and the b* value represents yellowness (+ b*) to blueness (− b*). A silicone putty jig with a 2 × 4 mm window (equal to the area of the exposed enamel) was fabricated on each block to allow for repeated measurements. One examiner performed all the measurements using a spectrophotometer (Minolta Chromameter CR- 241, Minolta Camera Co., Osaka, Japan) three times for each specimen at each time period over a gray background (L^*^ = 49.2, a^*^ = −0.4, b^*^ = 0.0) and recorded the mean values. The color difference (ΔE) between 2 stages was calculated using the following equation: ΔE = $\surd$(ΔL*)^2^ + (Δa*)^2^ + (Δb*)^2^, where ΔL*, Δa*, and Δb* represent changes in lightness, red-green coordinate, and yellow-blue coordinate, respectively [5]. ΔE and ΔL were calculated after demineralization (ΔE and ΔL_demineralization-baseline_), staining (ΔE and ΔL_SDF-demineralization_), after 2-week intervention (ΔE and ΔL_2-week intervention-staining_), and after 3-week intervention (ΔE and ΔL_3-week intervention-staining_).

### Statistical analysis

All measurements are represented as mean value ± the standard deviation (± SD) of the mean using SPSS version 22.0 (IBM Corp, Armonk, NY, USA) software. The EMH, ΔE, and ΔL values, except for the 3-week intervention data, were analyzed with one-way ANOVA and the Tukey’s post-hoc test. The paired t-test was used to compare the EMH, %REMH, ΔE, and ΔL values of Groups 2 and 4 at the end of the exposure to bleaching material. Besides, the paired t-test was used to compare %REMH between two time points. The significance level was set at *p* < 0.05.

## Results

Table 1 shows the mean ± SD for EMH in each group at different times. At the baseline, EMH ranged from 304.46 to 358.7 VHN (mean: 333.25 ± 14.06 VHN) with no significant differences among the groups (*p* = 0.998). The demineralizing solution significantly reduced EMH values in all groups (all, *p* < 0.00001). EMH values were not statistically different among groups after demineralization (*p* > 0.05). Two weeks after SDF or SDF + KI application, EMH values dramatically increased in all groups as compared to the demineralized status (all, *p* < 0.00001). EMH values were not significantly different among groups after SDF or SDF + KI application (*p* > 0.05). Notably, EMH values at this stage did not show significant difference from the baseline values (*p* = 0.327). Neither 2-week nor 3-week exposure to bleaching material did not cause significant decrease in EMH values compared to both the s-RCLs and the baseline status (all, *p* > 0.05).

**Table 1:** Comparison of enamel surface microhardness in experimental groups

Group	Baseline (mean ± SD)	Dem (Mean ± SD)	SDF/SDF + KI (mean ± SD)	2-week int (mean ± SD)	3-week int (mean ± SD)
1	333.75 ± 15.46^A,a^	230.01 ± 11.31^A,b^	337.52 ± 13.82^A,a^	332.74 ± 15.16^A,a^	
2	333.44 ± 15.37^A,a^	230.60 ± 11.29^A,b^	338.34 ± 9.84^A,a^	333.82 ± 11.12^A,a^	327.93 ± 12.40^A,a^
3	333.28 ± 14.32^A,a^	230.75 ± 11.55^A,b^	337.19 ± 13.06^A,a^	333.19 ± 12.24^A,a^	
4	332.51 ± 15.15^A,a^	230.87 ± 11.28^A,b^	337.00 ± 10.95^A,a^	332.76 ± 10.42^A,a^	327.66 ± 13.88^A,a^
*p* value	0.998	0.998	0.993	0.996	0.436

In each row, means with the same lowercase letter are not significantly different (within-group analysis)

In each column, means with the same capital letter are not significantly different (between-group analysis)

Statistical significance: *p* < 0.05

Group 1: SDF-treated enamel followed by 8-h/day application of 10% CP for 2 weeks; Group 2: SDF-treated enamel followed by 15-min/day application of 10% CP for 3 weeks; Group 3: SDF + KI-treated enamel followed by 8-h/day application of 10% CP for 2 weeks; and Group 4: SDF + KI-treated enamel followed by 15-min/day application of 10% CP for 3 weeks

*Dem.* demineralization, *SDF* silver diamine fluoride, *KI* potassium iodide, *int* intervention, *SD* standard deviation

To better compare the EMH values at different stages, the percentage recovery of enamel microhardness (%REMH) was calculated using the formula explained previously. Interestingly, all intervention groups showed complete recovery of EMH values after SDF application compared to demineralization (%REMH_SDF_) (*p* > 0.05). Bleaching caused a slight decrease in %REMH for all groups. However, the differences were not statistically significant (*p* > 0.05). Table 2 demonstrates the mean ± SD for %REMH in each group at different periods.

**Table 2:** Comparison of percentage recovery of enamel microhardness (%REMH)

Group	%REMH_SDF_ (mean ± SD)	%REMH_2-week int_ (mean ± SD)	%REMH_3-week int_ (mean ± SD)
1	104.42 ± 8.77^A,a^	99.51 ± 12.38^A,a^	
2	106.71 ± 19.42^A,a^	101.15 ± 15.72^A,a^	94.44 ± 15.15^A, a^
3	104.52 ± 10.81^A,a^	100.51 ± 8.38^A,a^	
4	106.01 ± 14.34^A,a^	101.63 ± 13.14^A,a^	96.28 ± 13.64^A, a^
*p* value	0.971	0.979	0.757

In each row, means with the same lowercase letter are not significantly different (within-group analysis)

In each column, means with the same capital letter are not significantly different (between-group analysis)

Statistical significance: *p* < 0.05

Group 1: SDF-treated enamel followed by 8-h/day application of 10% CP for 2 weeks; Group 2: SDF-treated enamel followed by 15-min/day application of 10% CP for 3 weeks; Group 3: SDF + KI-treated enamel followed by 8-h/day application of 10% CP for 2 weeks; and Group 4: SDF + KI-treated enamel followed by 15-min/day application of 10% CP for 3 weeks

*%REMH* percentage recovery of enamel microhardness, *SDF* silver diamine fluoride, *int* intervention, *SD* standard deviation

**Fig. 1:**
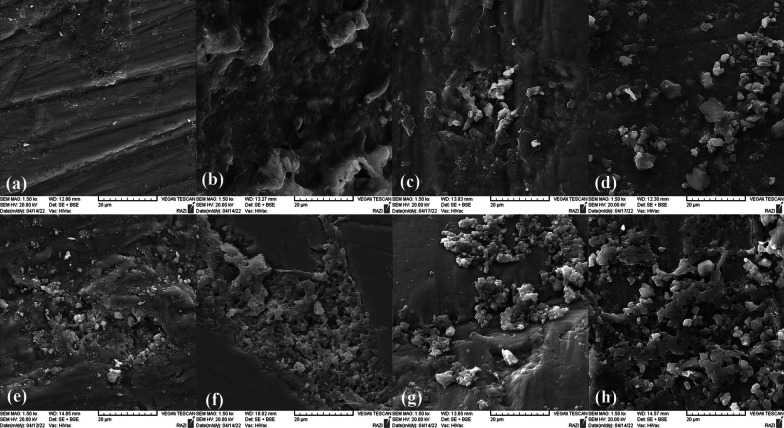
Scanning electron microscopy (SEM) images of the primary tooth enamel. **a** at the baseline; **b** after demineralization. An increase in the porosities and spaces in the enamel surface is evident; **c** aged remineralized-stained enamel (SDF-treated surface); **d** aged remineralized-stained enamel (SDF + KI-treated surface). The globular arrangement of mineral deposits has filled the porous areas and voids of the demineralized enamel; **e**–**h** after final intervention. Note that CP did not cause significant changes to the enamel surface. **e** SDF-treated enamel followed by 8-h/day application of 10% CP for 2 weeks; **f** SDF-treated enamel followed by 15-min/day application of 10% CP for 3 weeks; **g** SDF + KI-treated enamel followed by 8-h/day application of 10% CP for 2 weeks; **h** SDF + KI-treated enamel followed by 15-min/day application of 10% CP for 3 weeks

The effects of SDF and bleaching material application on the primary tooth enamel are presented in SEM images (Fig. 1a–h). Demineralization increased the porosities and spaces in the enamel surface due to dissolved minerals and organic materials (Fig. 1b). SEM images after SDF application (with and without KI) revealed agglomeration of mineral deposits with the irregular surfaces (Fig. 1c, d). Mineral precipitates, silver and fluoride-containing compounds, could nicely fill the porous areas and voids of the demineralized enamel. No microsurface alteration was evident after the bleaching process with 10% carbamide peroxide (Fig. 1e–h). However, the dissolution of the precipitated minerals in the enamel porosities was more evident in Groups 1 and 3 (Fig. 1e, g).

All samples were lighter in color after demineralization compared to their baseline color (Table 3). However, ΔE and ΔL were not significantly different among groups (*p* = 0.565 and *p* = 0.634, respectively). SDF application caused significantly darker colors in all samples (*p* < 0.00001). Groups 1 and 2 showed more severe changes in color than Groups 3 and 4 (all, *p* < 0.00001). No significant difference was observed between ΔE_SDF-Demin_ and ΔL_SDF-Demin_ of Groups 1 and 2 (*p* = 0.998 and *p* = 0.989, respectively) and Groups 3 and 4 (both, *p* = 0.998). Two-week exposure to bleaching material ameliorated the discoloration in all groups (all, *p*** < **0.00001). Group 3 and 4 had significantly lighter colors than group 1 and 2 after a 2-week intervention (all, *p*** < **0.00001). No significant change was observed between ΔE_2-week int-SDF_ of Groups 1 and 2 (*p* = 0.249) and Groups 3 and 4 (*p* = 0.979). ΔL_2-week int-SDF_ values of Groups 1 and 2 (*p* = 0.175) and Groups 3 and 4 (*p* = 0.967) showed no statistically significant differences. Groups 2 and 4 were exposed to the bleaching material for 3 weeks. All samples in Groups 2 and 4 had significantly lighter color after 21 days as compared to their 14-day exposure to the bleaching material (both, *p* < 0.00001). The changes in ΔE and ΔL values after the 3-week exposure to bleaching material showed significant differences among Groups 2 and 4 (both, *p* < 0.00001). The exemplary photos of the samples at different stages are illustrated in Additional file 1: Figure S1.

**Table 3:** Color change (ΔE) and brightness change (ΔL) mean ± standard deviation (SD) at different stages

ΔE	Group 1 (mean ± SD)	Group 2 (mean ± SD)	Group 3 (mean ± SD)	Group 4 (mean ± SD)	*p* value
ΔE_Dem-Base_	4.99 ± 1.38^a^	4.22 ± 1.57^a^	4.29 ± 1.64^a^	4.85 ± 1.86^a^	0.565
ΔL_Dem-Base_	4.15 ± 1.48^a^	3.48 ± 1.29^a^	3.73 ± 1.60^a^	4.20 ± 1.84^a^	0.634
ΔE_SDF-Dem_	28.14 ± 2.15^a^	28.09 ± 1.64^a^	16.03 ± 0.88^b^	15.82 ± 1.32^b^	< 0.00001
ΔL_SDF-Dem_	− 26.14 ± 2.34^a^	− 25.86 ± 2.24^a^	− 14.13 ± 2.02^b^	− 13.98 ± 2.25^b^	< 0.00001
ΔE_2-week int-SDF_	24.21 ± 1.68^a^	25.55 ± 1.44^a^	32.29 ± 1.93^b^	32.57 ± 1.86^b^	< 0.00001
ΔL_2-week int-SDF_	23.36 ± 1.89^a^	24.91 ± 1.54^a^	32.01 ± 1.76^b^	32.35 ± 2.05^b^	< 0.00001
ΔE_3-week int-SDF_		29.11 ± 1.57^a^		39.24 ± 1.77^b^	< 0.00001
ΔL_3-week int-SDF_		28.55 ± 1.92^a^		39.07 ± 1.89^b^	< 0.00001
ΔE_3-week-2-week int_		4.27 ± 0.91^a^		7.02 ± 0.61^b^	< 0.00001
ΔL_3-week-2-week int_		3.64 ± 88^a^		6.72 ± 0.84^b^	< 0.00001

In each row, means with the same lowercase letter are not significantly different (within-group analysis)

Statistical significance: *p* < 0.05

Group 1: SDF-treated enamel followed by 8-h/day application of 10% CP for 2 weeks; Group 2: SDF-treated enamel followed by 15-min/day application of 10% CP for 3 weeks; Group 3: SDF + KI-treated enamel followed by 8-h/day application of 10% CP for 2 weeks; and Group 4: SDF + KI-treated enamel followed by 15-min/day application of 10% CP for 3 weeks

## Discussion

In this study, we evaluated the impact of time of exposure of artificially created metallic s-RCLs of enamel surface to fluoridated bleaching material on EMH, surface topography, and color. We created initial caries-like lesions on the enamel surface as the porous structure of carious lesions are more prone to chromogen penetration than the sound enamel.

Determination of EMH is a non-destructive easy technique that has been frequently used to assess the impact of bleaching procedures on the enamel surface. All samples of our study had baseline EMH ranging from 304.46 to 358.7 VHN. The sound enamel specimens have surface hardness between 250 and 360 VHN [27]. We artificially created initial caries lesions. As a result, EMH values of all samples dramatically reduced compared to baseline, due to mineral loss from the enamel. Based on our results, the application of SDF (with or without KI) completely recovered the EMH values to the baseline level. According to the literature, SDF-arrested caries lesions have the same hardness values as the sound enamel [28] and are twice as hard as the sound dentin [29]. The main mechanism of SDF-induced increased hardness can be explained by the formation of silver phosphate (Ag_3_PO_4_), calcium fluoride (CaF_2_), and fluorapatite [Ca_10_(PO_4_)_6_F_2_] on treated lesions [30]. We kept the SDF-treated samples in deionized water for 2 weeks to create s-RCLs. It is suggested that caries lesion starts to arrest and reharden by 2 weeks [29, 31]. Existing literature reports controversial bleaching effects on surface microhardness, surface porosity, and susceptibility to further demineralization [7–10, 32, 33]. Based on our results, neither 2-week nor 3-week intervention did not cause significant decrease in EMH values compared to the baseline status. This finding is in line with previous studies [9, 10, 32]. The differences in study variables (composition, concentration, and duration of application of the bleaching material) and evaluation methods can explain the controversies in results [7]. Besides, we used fluoridated CP, which according to the manufacturer, contains 0.11% fluoride ion [13]. Fluoridated bleaching materials were previously reported to maintain EMH [13, 14].

We noted the globular arrangement of mineral deposits and an increase in the mineral density of the demineralized enamel in SEM images. As a water-soluble solution with a specific gravity of 1.35, SDF can be dissolved in the trapped water of enamel and dentin. The molecular and osmotic differences between water and SDF can pull SDF through the surface porosity [30]. The depth of silver and fluoride ions penetration is deeper in primary teeth (with an average depth of 744 µm) than the permanent ones (25–200 microns) [30]. This penetration potential might explain the increase in the microhardness after SDF treatment. Although the acidic nature of CP (pH 6.5) caused some dissolution of the precipitated minerals from the enamel surface, SEM analysis revealed no changes in enamel porosity after bleaching, which is in line with other studies [9–11]. Possible explanations for the conflicting results [8, 12] are the differences in the bleaching material with less pH levels than carbamide peroxide, and the differences in the methodology. We applied carbamide peroxide on SDF-treated carious lesions with evidently less porous areas than the demineralized enamel surface. Besides, fluoridated carbamide peroxide can cause further remineralization [13]. The longer period of CP application in Groups 1 and 3 can explain the minor differences in the surface mineral precipitates.

We used 38% SDF, which is composed of 24–27% silver, 8.5–10% ammonia, and 5–6% fluoride with a highly alkaline pH (pH 12.5) [34]. Despite its benefits, SDF application causes dark stains, which limits its clinical use [5]. In our study, all samples became significantly darker after SDF application, as indicated by negative ΔL_SDF-Demin_ values (Table 3). These results implied the successful incorporation of the metallic stains in the created lesions. In line with our findings, the immediate use of potassium iodide (KI) after the application of SDF has been suggested to minimize this adverse effect [6].

ΔE* values greater than 3.3 correlates with clinically visible changes in tooth color [23]. In this regard, all samples demonstrated a significant whitening effect after the 2-week exposure to bleaching material. This effect was more remarkable in KI-treated samples (Groups 3 and 4). The 3-week exposure to bleaching material resulted in visible changes in tooth color in both Groups 2 and 4. This effect was more remarkable in Group 4. This result corroborates previous studies suggesting that longer enamel bleaching time caused better color improvement regardless of the concentration of bleaching agents [7, 11].

There are few studies, mostly case reports, to evaluate the use of bleaching agents in primary dentition [15–20]. Determination of the appropriate duration of bleaching procedure more suitable for children was suggested by Lee et al. [17]. We found that both 8-h and 15-min application of the bleaching material could effectively lighten the metallic staining. This finding might be explained by the porous structure of the caries-induced enamel and the acidic pH of the bleaching material, which might help better penetration to tooth structure. Besides, primary tooth enamel has a higher interprismatic fraction (interprismatic area related to intraprismatic area) than its permanent counterpart [35]. It implies that primary tooth enamel is more porous and permeable than the permanent enamel [35].

The latest revision of American Academy of Pediatric Dentistry policy has considered adult/dentist-supervised bleaching procedure as a safe and beneficial modality for children and adolescents [21]. The General Dental Council (GDC) in its 2016 position statement on tooth whitening stated that products containing or releasing between 0.1 and 6% hydrogen peroxide cannot be used in any person under 18 years of age, “*except where such use is intended wholly for the purpose of treating or preventing disease”* [36]*.* Noteworthy, ten percent carbamide peroxide falls within the permitted range as it equivalents to 3 percent hydrogen peroxide [21]. Despite the GDC announcement, the use of bleaching products containing more than 0.1% hydrogen peroxide in patients under 18 years of age in countries belonging to the European Union is illegal (EU Cosmetics Regulation directive 2011/84/EU) [37].

Despite the limitation of in vitro studies, we tried to imitate the oral cavity condition by thermocycling aging and accurately following the manufacturer's instructions for materials application. As we only wanted to focus on the effect of SDF and bleaching materials on EMH, we did not preserve the samples in saliva. The absence of enough studies in primary teeth that have assessed the efficacy of bleaching materials precluded appropriate comparisons. For this reason, we compared our results with studies performed on permanent teeth. Besides, we did not extend our microscopic evaluation to measure the depth of penetration and changes in mineral content, which is another limitation. Initial grinding of enamel surface removes its aprismatic enamel and changes optical properties, and the use of unground specimens is desirable for correlating the results to daily practice. However, the unground enamel has surface irregularity and curvature, which causes a wide range of variability in the measurements. Therefore, we initially polished the enamel surface to provide a standardized surface, which helps reduce the biological sample variations. Further experimental and clinical studies in primary dentition are highly suggested to find effective and safe methods to eliminate metallic stains in primary teeth.

## Conclusion

Within the limitations of this in vitro study, we concluded that SDF could highly remineralize the demineralized enamel surface, and the SDF-treated surfaces were highly resistant to further acidic challenges due to bleaching material application. The use of fluoridated CP could effectively improve the color change caused by SDF application (with and without KI). Color improvement was more evident with the use of KI immediately after the SDF application. As both 15-min and 8-h application of fluoridated CP resulted in statistically similar color enhancement in primary teeth, a shorter duration of bleaching material application is highly acknowledged in pediatric patients.

## Supplementary Information

**Additional file 1. Figure S1:** Tooth samples: (a) SDF-treated enamel, (b) SDF+KI-treated enamel, (c) SDF-treated enamel followed by 8-hour/day application of 10% CP for two weeks; (d) SDF-treated enamel followed by 15-min/day application of 10% CP for three weeks; (e) SDF+KI-treated enamel followed by 8-hour/day application of 10% CP for two weeks; (f) SDF+KI-treated enamel followed by 15-min/day application of 10% CP for three weeks

## Data Availability

The datasets generated during and/or analyzed during the current study are available from the corresponding author upon reasonable request.

## Abbreviations

CP: Carbamide peroxide
KI: Potassium iodide
SDF: Silver diamine fluoride
s-RCLs: Stained-remineralized caries lesions
EMH: Enamel microhardness
SEM: Scanning electron microscopy
%REMH: Percentage recovery of enamel microhardness
APF: Acidulated phosphate fluoride
VHN: Vickers hardness number
