# Supplementary figures and images for: Evaluation of bleaching agent effects on color and microhardness change of silver diamine fluoride-treated demineralized primary tooth enamel: An in vitro study
Source: BMC Oral Health. 2022 Aug 12;22:347. doi: 10.1186/s12903-022-02371-3 (PMC9373438; doi:10.1186/s12903-022-02371-3)

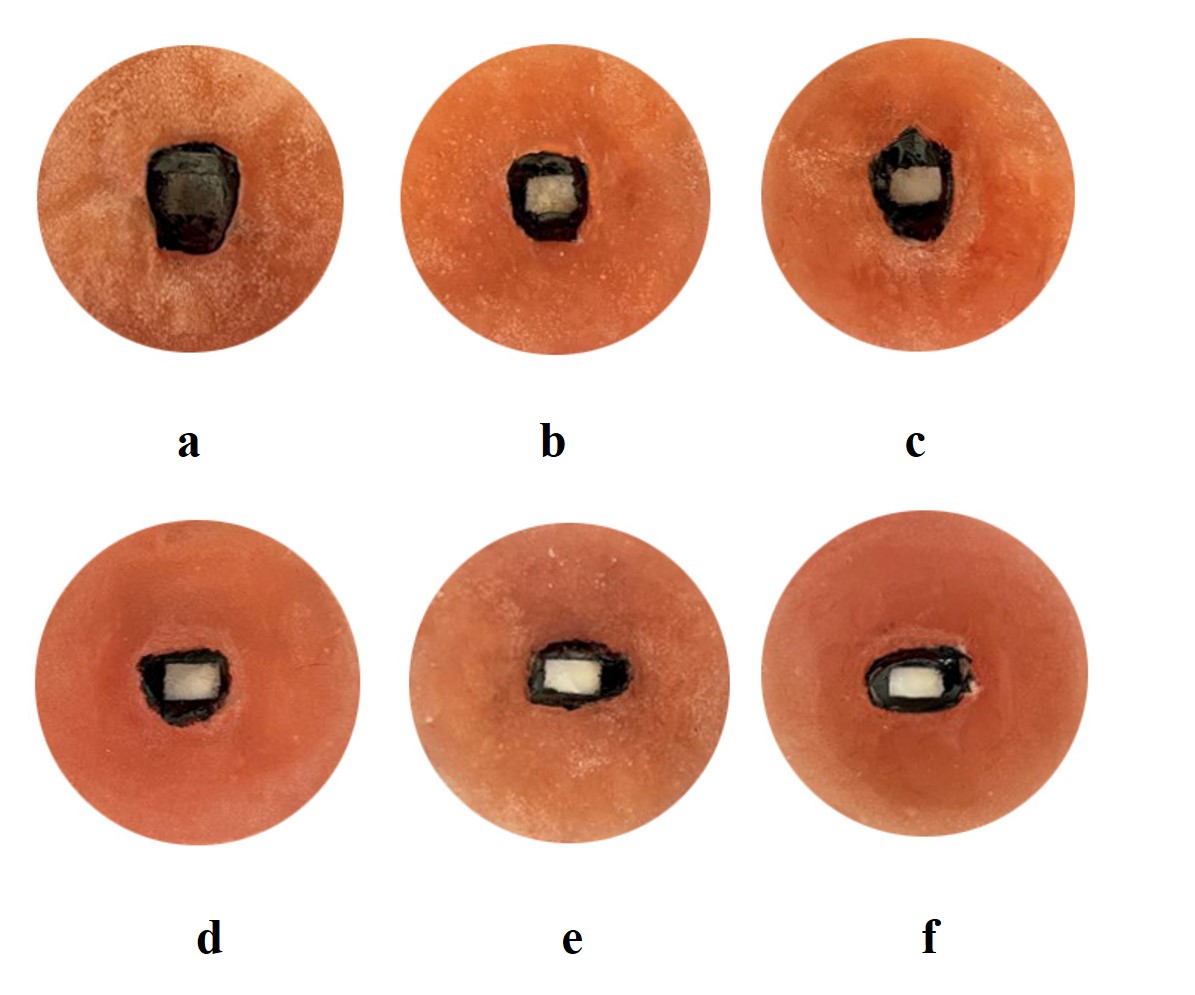

Supplement: Supplementary file 1 — Additional file 1. Figure S1: Tooth samples: (a) SDF-treated enamel, (b) SDF+KI-treated enamel, (c) SDF-treated enamel followed by 8-hour/day application of 10% CP for two weeks; (d) SDF-treated enamel followed by 15-min/day application of 10% CP for three weeks; (e) SDF+KI-treated enamel followed by 8-hour/day application of 10% CP for two weeks; (f) SDF+KI-treated enamel followed by 15-min/day application of 10% CP for three weeks [file 12903_2022_2371_MOESM1_ESM.jpg]
